# Structural Characterization of Cardiac Free‐Running Purkinje Fibers Using Inhomogeneous Magnetization Transfer (ihMT): A Proof of Concept MRI‐Histology Approach

**DOI:** 10.1002/nbm.70289

**Published:** 2026-04-19

**Authors:** Arash Forodighasemabadi, Evgenios N. Kornaropoulos, Marion Constantin, Lucas Soustelle, Fanny Vaillant, Jude Leury, Richard Walton, Olivier Bernus, Bruno Quesson, Olivier M. Girard, Guillaume Duhamel, Julie Magat

**Affiliations:** ^1^ Univ. Bordeaux, INSERM, CRCTB, U 1045, IHU Liryc Bordeaux France; ^2^ Aix Marseille Univ, CNRS, CRMBM Marseille France; ^3^ CRC Human Imaging, GIGA Department University of Liege Liege Belgium; ^4^ APHM, Hôpital Universitaire Timone, CEMEREM Marseille France; ^5^ CRMSB UMR 5536 CNRS/Université de Bordeaux Bordeaux France

**Keywords:** 9.4 T MRI, cardiac conduction system, free‐running, histology, inhomogeneous magnetization transfer, myocardium, PMJ, Purkinje fiber

## Abstract

The cardiac Purkinje network plays a critical role in maintaining synchronized ventricular activation but remains difficult to image due to its fine and complex structure. Conventional MRI techniques lack sufficient contrast to distinguish the structural composition of Purkinje fibers (PFs). This study investigates the potential of inhomogeneous magnetization transfer (ihMT) as a novel contrast mechanism for visualizing and differentiating subregions of the Purkinje network. Five fixed ex vivo sheep hearts containing free‐running PFs were scanned using a 9.4 T MRI system with a 2D ihMT RARE sequence. ihMTR maps were analyzed using manually defined regions of interest (ROIs) corresponding to free‐running fibers, the Purkinje–myocardial junction (PMJ), and the surrounding myocardium. Histological analysis was performed on matched tissue sections to quantify collagen types I and III, adipocytes, Purkinje cells, and cardiomyocytes. Three ihMT protocols that produced high ihMTR values in free‐running fibers (9.25–10.83%) and strong contrast relative to myocardium (2.00–2.17%) and the PMJ (2.99–3.40%) in 1 sample were selected and applied to all samples. Across all hearts, mean ihMTR values were consistently higher in free‐running fibers compared to the PMJ (11.5 ± 1.5% vs 9.0 ± 2.9%). Histological analysis revealed significantly greater collagen content in free‐running regions compared with the PMJ (72.4 ± 15.9% vs 31.1 ± 13.1%; p = 0.001), along with higher adipocyte content at the PMJ compared to free‐running regions (12.3 ± 6.1% vs 3.8 ± 2.7%, not significant). Collagen type III was more prominent at the PMJ but remained a minor component overall. These findings demonstrate that ihMT imaging can distinguish PF subregions based on underlying microstructural differences, particularly collagen and adipocyte distribution. This study lays the groundwork for developing biophysical models to interpret ihMT signals in terms of tissue composition and microstructure, providing a foundation for future studies.

## Graphical abstract

The cardiac Purkinje network is essential for synchronized ventricular activation but remains difficult to visualize with conventional MRI due to limited structural contrast. This study demonstrates that inhomogeneous magnetization transfer imaging provides enhanced contrast that differentiates free‐running Purkinje fibers, Purkinje‐Myocardial Junction, and myocardium. These ihMT contrasts reflect underlying microstructural differences, particularly variations in collagen and adipocyte distribution, highlighting ihMT as a promising tool for Purkinje network imaging.

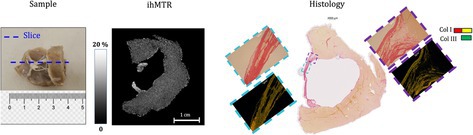

**Abbreviations**
CCS: cardiac conduction system
ihMT: inhomogeneous magnetization transfer
MT: magnetization transfer
NEX: number of excitations
PF: Purkinje fiber
PL: polarized light
PMJ: Purkinje–myocardial junction
SAR: specific absorption rate

## 1 Introduction

The His–Purkinje system is a specialized network of fibers that plays a critical role in the synchronous activation of the ventricles by providing a rapid conduction pathway [1]. This coordination ensures efficient ventricular contraction, which is essential for maintaining proper cardiac function [2]. However, the Purkinje system is also implicated in the initiation and maintenance of ventricular fibrillation, which can lead to sudden cardiac death [3]. Understanding this intricate system is therefore crucial for preventing and treating cardiac arrhythmias [4].

From a structural point of view, Purkinje cells, the cellular component of the His–Purkinje network, are considerably larger than myocardial cells in their transverse section size [5]. They also exhibit morphological differences across species; for instance, ungulates have large, clearly differentiated Purkinje fibers (PFs) with few peripheral myofibrils and abundant central glycogen, whereas rodents possess PFs that are structurally very similar to working myocardial cells [6]. In ungulates like sheep and pigs, PFs within false tendons are surrounded by a dense collagen sheath. This sheath is thought to act as an electrical insulator and likely contributes to the rapid and efficient conduction of the action potential and may also provide mechanical support and protection to these specialized conducting cells [2, 6]. There are also structural differences within the fiber itself, notably between the free‐running part surrounded by collagen and optimized for rapid conduction and the Purkinje–myocardial junction (PMJ), which are responsible for transmitting impulses to the slower‐conducting myocardial tissue [7].

Recent advancements in imaging technologies have significantly improved the study of the His–Purkinje system. Micro‐computed tomography (micro‐CT), enhanced with iodine‐based contrast agents, has facilitated high‐resolution visualization of the cardiac conduction system (CCS) [8, 9, 10]. For instance, Stephenson et al. used contrast‐enhanced micro‐CT to distinguish major subdivisions of the CCS in rat and rabbit hearts, including the Purkinje network [11]. Later, this method was employed to generate the first 3D representation of the human CCS [8] and to examine morphological changes in pathological hearts [10]. Similarly, Aminu et al. utilized micro‐CT with graphene oxide contrast agents to reconstruct the CCS in fixed human hearts [9], and Chen et al. used contrast‐enhanced CT to study anatomical changes in Myocardial Infarction [12].

In contrast to the CT, magnetic resonance imaging (MRI) is a nonionizing modality that can provide 2D and 3D images in any orientation, with contrasts that can be adjusted depending on the acquisition technique. However, the required spatial resolution to visualize the 3D morphology of conduction pathways remains a major challenge. Hwang et al. used high‐resolution MRI to trace conduction pathways in isolated rabbit hearts, demonstrating its capability to map the Purkinje‐ventricular junctions in 3D [13]. However, conventional MRI techniques, based on relaxation properties (*T*_1_, *T*_2_, and *T*_2_*) and proton density, often lack sufficient contrast for effectively differentiating PF from the surrounding cardiac muscle [14]. An ex vivo study on swine hearts has shown that rotating frame relaxation maps can provide contrast between the atrioventricular conduction axis and myocardium, demonstrating the potential of quantitative MRI techniques for characterizing the conduction system [15].

Magnetization transfer (MT) has demonstrated sensitivity to highly organized macromolecules, including collagen. Several studies on phantoms [16] and biological tissues such as intervertebral discs [17], knee cartilage [16], renal [18] and liver fibrosis [19], and myocardial scars [20] have shown that the MT signal correlates with collagen content. Magat et al. [14] explored MT contrast for imaging the Purkinje network and provided optimized sequence parameters to acquire 3D images of the PFs in ex vivo samples of porcine heart.

Inhomogeneous magnetization transfer (ihMT) [21, 22, 23, 24] is an extension of MT and is sensitive to dipolar order relaxation [23]. IhMT has been primarily applied to brain [22, 25] and spinal cord [26] imaging due to its high sensitivity and specificity to myelinated tissue [27], making it particularly useful for studying demyelinating pathologies such as multiple sclerosis [28]. Given the sensitivity of ihMT for tissues with highly organized structure and the known correlation between MT signal and collagen content, applying ihMT to CCS, particularly PF, which are rich in collagen, may hold potential for improved characterization of these structures. In this study, we present an experimental exploration of ihMT sequence parameters aimed at enhancing contrast for improved visualization of free‐running PF in ex vivo cardiac samples. MR imaging findings are complemented by histological analysis to support tissue characterization.

## 2 Materials and Methods

### 2.1 Experimental Setup

**FIGURE 1:**
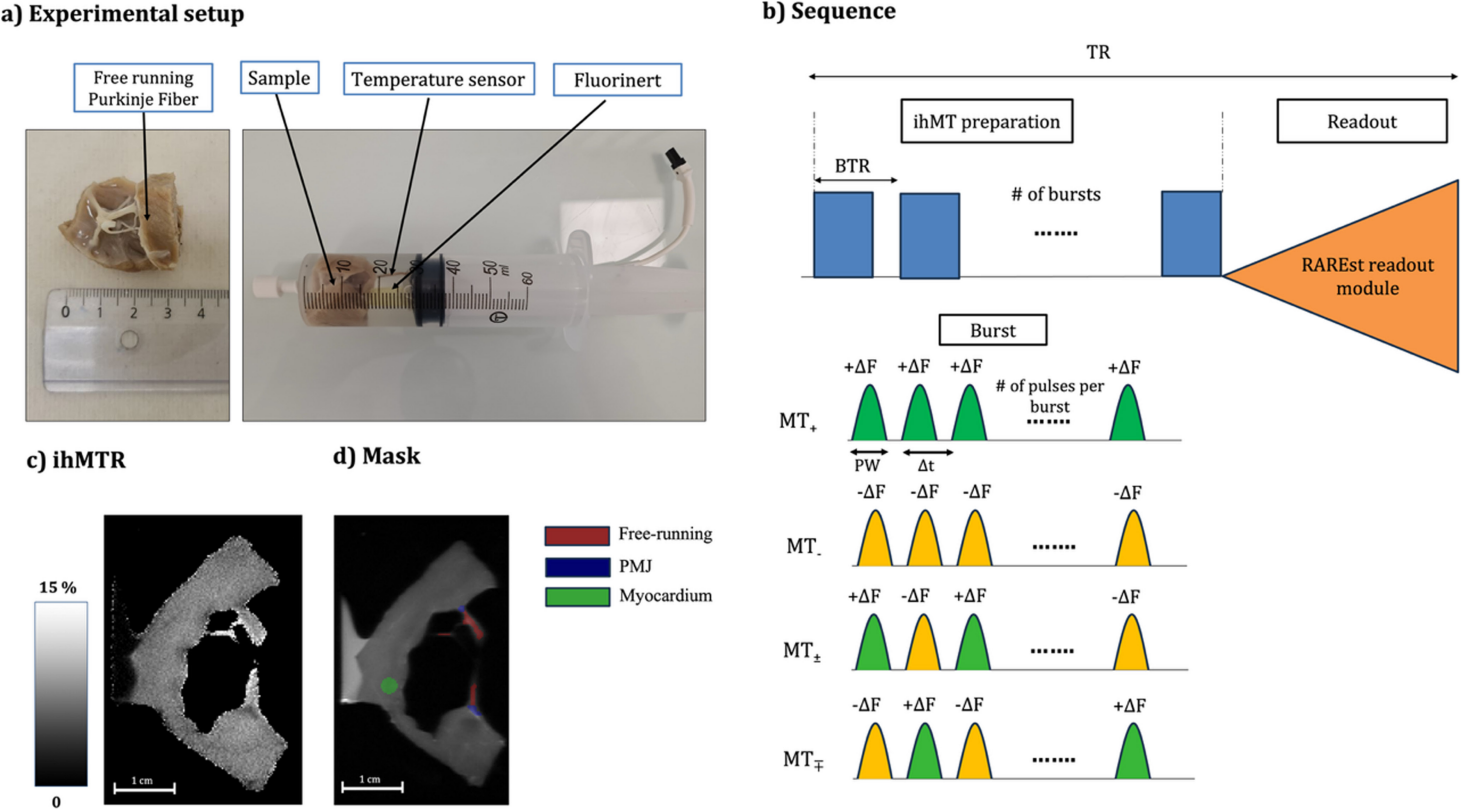
(a) Experimental setup showing the sample containing myocardium and PF placed in a syringe filled with Fluorinert, equipped with a temperature probe connected to an external computer for monitoring. (b) 2D ihMTRARE sequence, featuring ihMT preparation with bursts of MT pulses followed by readout (PW: pulse width; ∆*t*: repetition delay; BTR: burst TR). (c) An example of ihMTR map in %. (d) The mask on *M*_0_ including free‐running PF (red), PMJ (blue), and myocardium (green).

Five samples (S1–S5), all taken from the left ventricle and containing myocardium along with free‐running PF (Figure 1s), were obtained from the hearts of five female sheep (age = 9 ± 0.7 years, weight = 50.5 ± 6.5 kg) following euthanasia via sternal thoracotomy under general anesthesia. The animal protocol has been described in Cabanis et al. [29]. The tissues were fixed in 4% formaldehyde containing 0.1% gadoterate meglumine (0.5 mmol/mL; Dotarem, Guerbet, France). The animal protocol was approved by the Animal Research Ethics Committee (CEEA50) in accordance with the European rules for animal experimentation (European legislation 2010/63 EU). For MRI data acquisition, each sample (approximate size of S1: 35 × 35 × 25 mm^3^, S2: 55 × 35 × 35 mm^3^, S3: 30 × 25 × 35 mm^3^, S4: 35 × 30 × 30 mm^3^, S5: 35 × 25 × 30 mm^3^) was placed in a syringe filled with Fluorinert (a liquid that does not produce a signal in proton MR; CF2, Sigma‐Aldrich, St. Louis, MO) (Figure 1a) to mitigate potential susceptibility artifacts. The syringe was equipped with a temperature probe (SA Instruments, NY) connected to a computer for continuous monitoring of temperature during the experiments (Figure 1a). The samples were heated using water bath tubing wrapped around the syringe.

In the first phase of the study, termed *protocol selection*, one sample was heated to 33°C ± 1°C and subjected to a range of ihMT preparation saturation parameters to identify optimal contrast settings. During the ihMT preparation, parameters such as pulse width, *B*_1_ amplitude, number of pulses, and offset frequency (Figure 1b) were systematically varied, as they are known to significantly influence ihMT signal and contrast. In the second phase, termed *protocol application*, three optimized protocols selected from the first phase were applied on all five samples, heated to 36°C ± 1°C.

### 2.2 MR Acquisition

All MR experiments were performed at 9.4 T using a BioSpec 94/30 scanner (Bruker BioSpin MRI, Ettlingen, Germany) equipped with a cylindrical transmit coil (200‐mm internal diameter) and a 4‐channel phased‐array receive coil and equipped with gradients with a maximum 300‐mT/m strength.

After acquiring the localizers, GRE sequences (in axial, coronal, and sagittal views) were run to identify the best slice for visualizing both the PF and the myocardium.

A 2D ihMT‐prepared centric‐out single‐shot RARE sequence (Figure 1b) was used. The ihMT preparation consisted in the acquisition of four MT‐weighted images obtained from different saturation preparations. These include positive single‐sided (MT_+_), positive–negative dual‐sided (MT_±_), negative single‐sided (MT_−_), and negative–positive dual‐sided (MT_∓_) images. Dual‐sided saturations were implemented using frequency‐alternated radiofrequency (RF) pulses that alternate between positive (+Δ*f*) and negative (−Δ*f*) frequency offsets.

During the protocol selection phase, the sample contained a free‐running fiber with a diameter of up to more than a millimeter. For this sample, partial Fourier acquisition (factor = 1.8 or 55% of *k*‐space acquired) was applied, enabling the collection of 200 volumes for each of the four MT‐weighted images within a total scan time of approximately 2 h. Other parameters included a TE of 20 ms, TR of 3000 ms, 10 averages of reference images without any preparation module (MT_0_), and a voxel size of 0.25 × 0.25 × 1 mm^3^.

In the protocol application phase, as the fibers of other samples were thinner, partial Fourier was omitted to minimize partial volume effects and blurring. The number of MT‐weighted volumes was reduced to 50 per image to maintain a comparable total acquisition time, whereas all other acquisition parameters remained the same.

### 2.3 MR Images Postprocessing

IhMT ratios (ihMTR, expressed in %) were calculated as

$$\text{ihMTR}=100\times \frac{{\mathrm{MT}}_{\text{sing}}-{\mathrm{MT}}_{\text{dual}}}{{\mathrm{MT}}_{0}}$$

with *MT*_*sing*_ = *MT*_+_+*MT*_−_ and *MT*_*dual*_ = *MT*_±_ + *MT*_∓_. IhMTR maps were generated using the publicly available script: https://github.com/lsoustelle/ihmt_proc (hash f3f49e0). An example of ihMTR map is shown in Figure 1c.

Three regions of interest (ROIs) were manually drawn using the ITK‐SNAP toolbox (www.itksnap.org) [30]. One ROI was placed over the free‐running segment of the fiber (Figure 1d, red), another at the PMJ where the fiber enters the myocardium (Figure 1d, blue), and a third within the myocardium (Figure 1d, green).

In the *protocol selection* phase of the study, the MT saturation parameters detailed in Table 1 were investigated on Sample 1. The contrast values (in ihMTR units) were defined as

$$\begin{array}{c} \text{Contrast}\left(\text{Free}-\text{running}\;\mathrm{vs}.\text{Myocardium}\right)=|\mathrm{ihM}TR_{\text{Free}-\text{running}}-{\text{ihMTR}}_{\text{Myocardium}}| \end{array}$$

$$\text{Contrast}\left(\text{Free}-\text{running}\;\mathrm{vs}.PMJ\right)=|{\text{ihMTR}}_{\text{Free}-\text{running}}-{\text{ihMTR}}_{PMJ}|$$

Based on the results obtained, three protocols listed in Table 2 were selected and used in the *protocol application* phase on all five samples.

**TABLE 1:** All ihMT sequence configurations were applied with Hann‐shaped pulses for a saturation B1RMS (defined over BTR) maintained constant at 9 ± 0.3 μT. As an example, based on the table, parameters for acquisition #12 are PW/∆*t*/#pulses per burst/#bursts/∆*F* = 1 ms/1.1 ms/12/13/120 ms/18 kHz and for acquisition #13 are PW/∆*t*/#pulses per burst/#bursts/∆*F* = 1 ms/3 ms/12/38/40 ms/14 kHz.

# Acquisition	PW (ms)	∆*t* (ms)	# Pulses per burst	# of bursts	BTR (ms)	∆*F* (kHz)	Duty cycle (%)	FA (°)
1–3	1	1.1	12	42	36	14; 20; 25	33.8	195
4–7				32	48	10; 14; 20; 25	25.5	225
8–10				19	80	10; 14; 18	15.6	285
11, 12				13	120	14; 18	10.7	345
13, 14		3		38	40		30.08	205
15, 16				32	48		25.19	225
17, 18				19	80		15.45	291
19, 20				13	120		10.57	356
21, 22	0.5	0.625	24	42	36		33.8	97.5
23, 24				32	48		25.5	112.5
25, 26				19	80		15.6	145
27, 28				13	120		10.7	178
29, 30			60	40	37.7		79.9	63
31, 32				25	60		50.7	79.5
33, 34				17	90		34.5	97.5
35, 36				13	120		26.3	112.5
37, 38				8	200		16.6	145
39, 40				6	300		11.71	145

Abbreviations: ∆*t*: repetition delay; BTR: burst TR; PW: pulse width.

**TABLE 2:** The three selected protocols providing the best contrast on Sample 1 during the *Protocol selection* phase.

Protocol	PW (ms)	∆*t* (ms)	# Pulses per burst	# Bursts	BTR (ms)	∆*F* (kHz)
Pr1	1	1.1	12	32	48	20
Pr2	0.5	0.625	24	32	48	18
Pr3	0.5	0.625	60	17	90	18

Abbreviations: ∆*t*: repetition delay; BTR: burst TR; PW: pulse width.

### 2.4 Histological Analysis

Following MR experiments, histological analyses were performed to provide a detailed characterization of tissue microstructure. After dehydration, samples were embedded in paraffin and sectioned at 6‐μm thickness. Three staining protocols were applied: Masson's trichrome, Periodic Acid Schiff (PAS), and Picro Sirius Red.

Masson's trichrome, which highlights cellular morphology, and PAS, which stains glycogen content in dark purple, were first used to confirm the presence of PFs in the free‐running regions. Picro Sirius Red staining was subsequently employed for structural identification, with collagen fibers, myocytes, and adipocytes appearing red, yellow, and white, respectively. Observation under polarized light (analyzer–polarizer, Nikon) enabled discrimination between collagen Type I (red, orange, and yellow) and collagen Type III (in green) [31]. For Samples S1–S5, 2, 2, 3, 4, and 2 histological sections with Picro Sirius Red staining, respectively, were selected as the closest anatomical matches to the corresponding ihMTR imaging slices. Slices were examined at 20× magnification on a tissue slide scanner (Axio Scan Z1, Carl Zeiss Microscopy GmbH, Jena, Germany) or at 4× magnification using an optical microscope (Eclipse 80i, Nikon, Tokyo, Japan).

### 2.5 Histological Images Postprocessing

Segmentation of adipocytes and collagen in Picro Sirius Red histology images was performed using ImageJ with manual color thresholding. The segmentation process involved adjusting hue, saturation, and lightness to isolate blue pixels corresponding to collagen and dark pixels corresponding to adipocytes. This approach enabled the creation of binary masks for both tissue components.

For collagen segmentation under polarized light, a home‐made Python program was written to convert RGB images (red–green–blue in PNG format) color space to HLS (hue–lightness–saturation) to facilitate color‐based classification [32]. The hue values were scaled from 0 to 360, and thresholding was applied based on the hue component to distinguish different colors within the stained collagen. Pixels with hue values between 330–359 and 0–20 were classified as red, 20–38 as orange, 39–61 as yellow, and 62–128 as green. The segmentation was applied to PMJ and free‐running regions of each sample. The proportion of each color was calculated as the number of pixels within each hue range, normalized to the total collagen content. The percentages of red, orange, and yellow pixels were summed to represent collagen Type I, whereas the percentage of green pixels was reported as collagen Type III.

## 3 Results

### 3.1 Protocol Selection Phase

**FIGURE 2:**
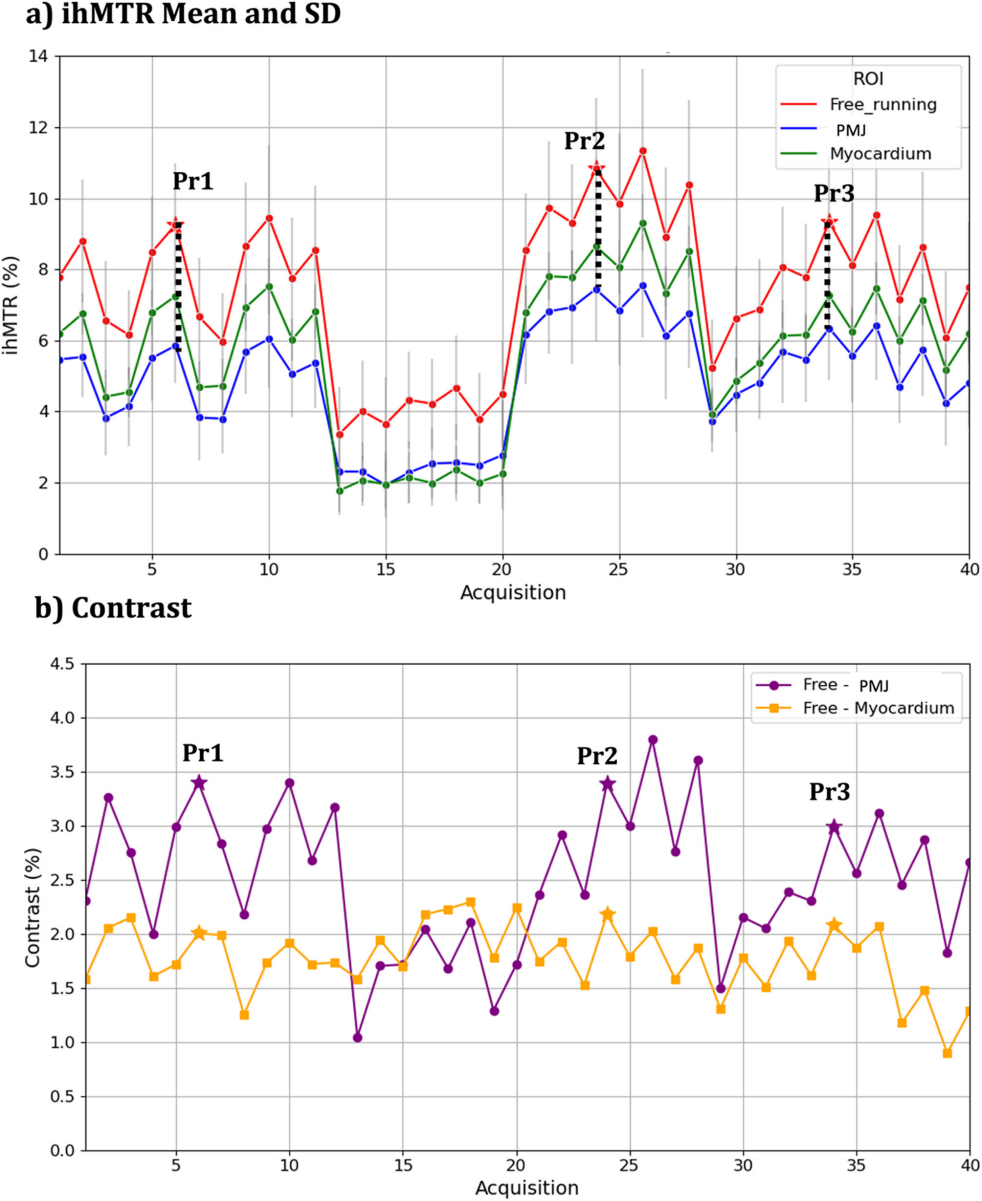
(a) IhMTR in % for free‐running PF (red), PMJ (blue), and myocardium (green) across all experiments. (b) Contrast (in ihMTR units) for free‐running PF vs. myocardium (orange) and for free‐running PF vs. PMJ (purple) across all experiments. The three protocols selected for *protocol application* phase are indicated on both figures.

Figure 2 presents mean ihMTR values calculated over the chosen ROIs (Figure 2a) and the associated contrasts between structures (Figure 2b) for different acquisition parameters during the protocol selection phase on Sample 1. Across all acquisitions, the ihMTR in free‐running is consistently higher than in the myocardium and the PMJ. Acquisition Protocols 13–20 (see Table 2 for parameter values) are excluded from analysis due to their lower baseline ihMTR values in the free‐running region (see Figure 2a). Among the remaining acquisitions, we selected three protocols representing different configurations of saturation pulse duration and number of pulses per burst. The selected protocols, referred to as Protocols 1, 2, and 3 (Pr 1, Pr 2, and Pr 3, respectively), exhibited high ihMTR values in the free‐running fiber (9.25%, 10.83%, and 9.33%, respectively in Figure 2a), along with strong contrast both between free‐running and myocardium (2.00%, 2.17%, and 2.07%, absolute ihMTR scale, in orange in Figure 2b) and between free‐running and the PMJ (3.40%, 3.39%, and 2.99% in purple in Figure 2b).

### 3.2 Protocol Application Phase

**FIGURE 3:**
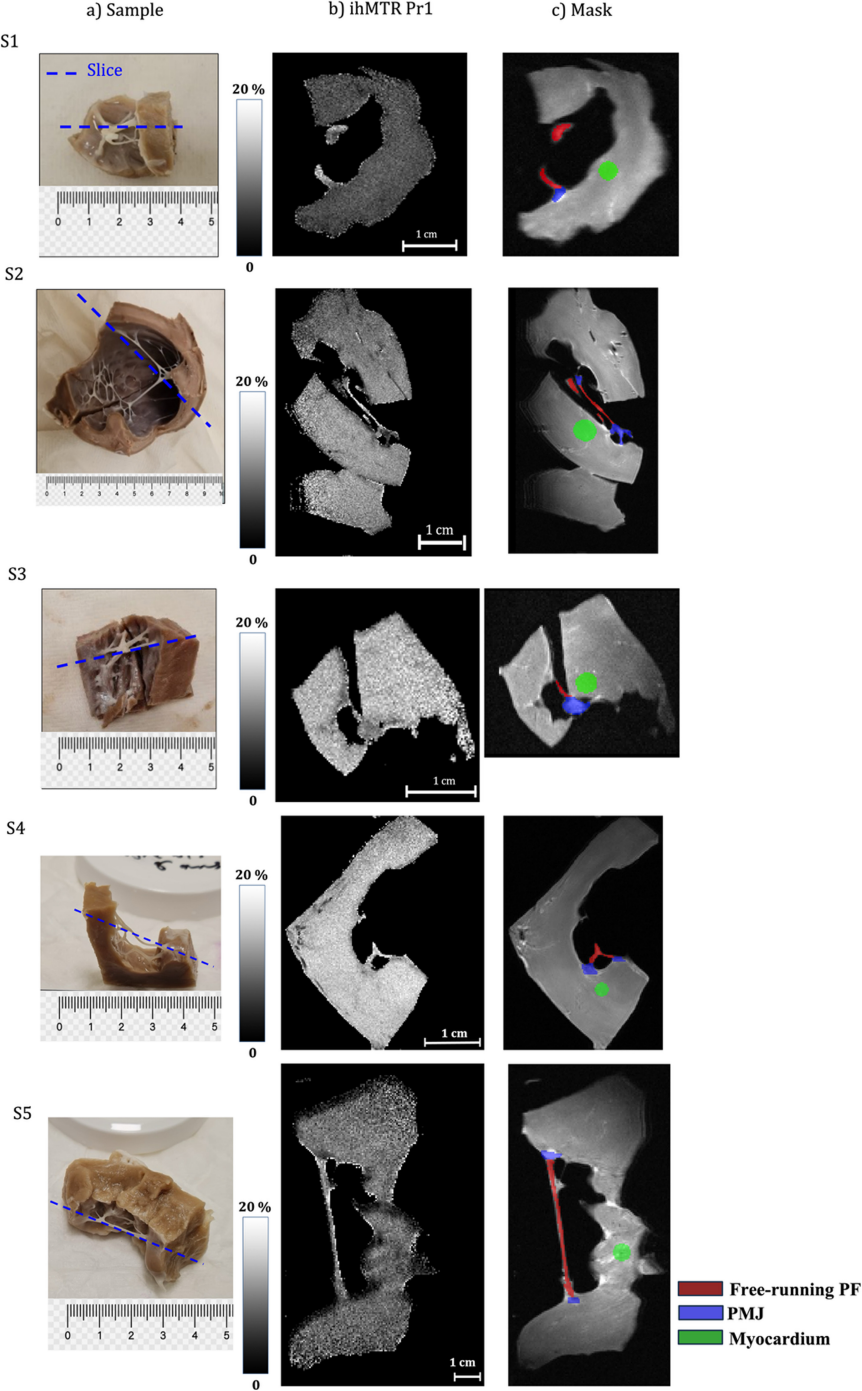
(a) Photos of each sample with a dashed blue line indicating the image slice location (all in axial orientation); (b) ihMTR from Protocol 1; (c) the mask used in the protocol application phase differentiating the fiber's PMJ (blue), the free‐running section (red), and the myocardium (green) for each sample.

Figure 3 presents (a) representative photographs of all samples used during the protocol application phase, together with the corresponding imaging slice, (b) ihMTR maps obtained using Protocol 1, and (c) the selected ROIs in the free‐running fibers (red), PMJ (blue), and myocardium (green). Overall, ihMTR values are higher in the free‐running regions of the PF compared with the PMJ, whereas myocardial contrast exhibits substantial intersample variability.

Detailed ihMTR mean ± SD values for each ROI, sample, and protocol are provided in Table S1 for interested readers. Table 3 summarizes the ihMTR (mean ± interprotocol SD), averaged across the three imaging protocols, for each ROI and for each of the five samples. Across all samples, ihMTR values are consistently (nonsignificantly) higher in the free‐running fiber region than at the PMJ (11.5% ± 1.5% vs. 9.0% ± 2.9%, respectively). In contrast, this trend was not observed in the myocardium: In Samples S2 and S3, myocardial ihMTR values exceed those measured in the free‐running regions (12.7% ± 1.2% vs. 11.9% ± 0.6% and 13.9% ± 1.1% vs. 11.0% ± 1.3% for myocardium vs. free‐running regions in S2 and S3, respectively).

**TABLE 3:** ihMTR (mean ± SD) averaged over three protocols in % for each ROI and each sample. The mean values are consistently higher in free‐running than in PMJ although not statistically significant (paired *t*‐test *p*‐value = 0.06).

# Sample	Free‐running	PMJ	Myocardium
1	10.6 ± 0.9	6.3 ± 0.6	8.1 ± 1
2	11.9 ± 0.6	7.7 ± 0.7	12.7 ± 1.2
3	11 ± 1.3	9.5 ± 0.2	13.9 ± 1.1
4	14.1 ± 1.4	13.8 ± 1.4	11 ± 1
5	10.2 ± 0.9	7.9 ± 0.8	6.5 ± 0.5

### 3.3 Histological Analysis

**FIGURE 4:**
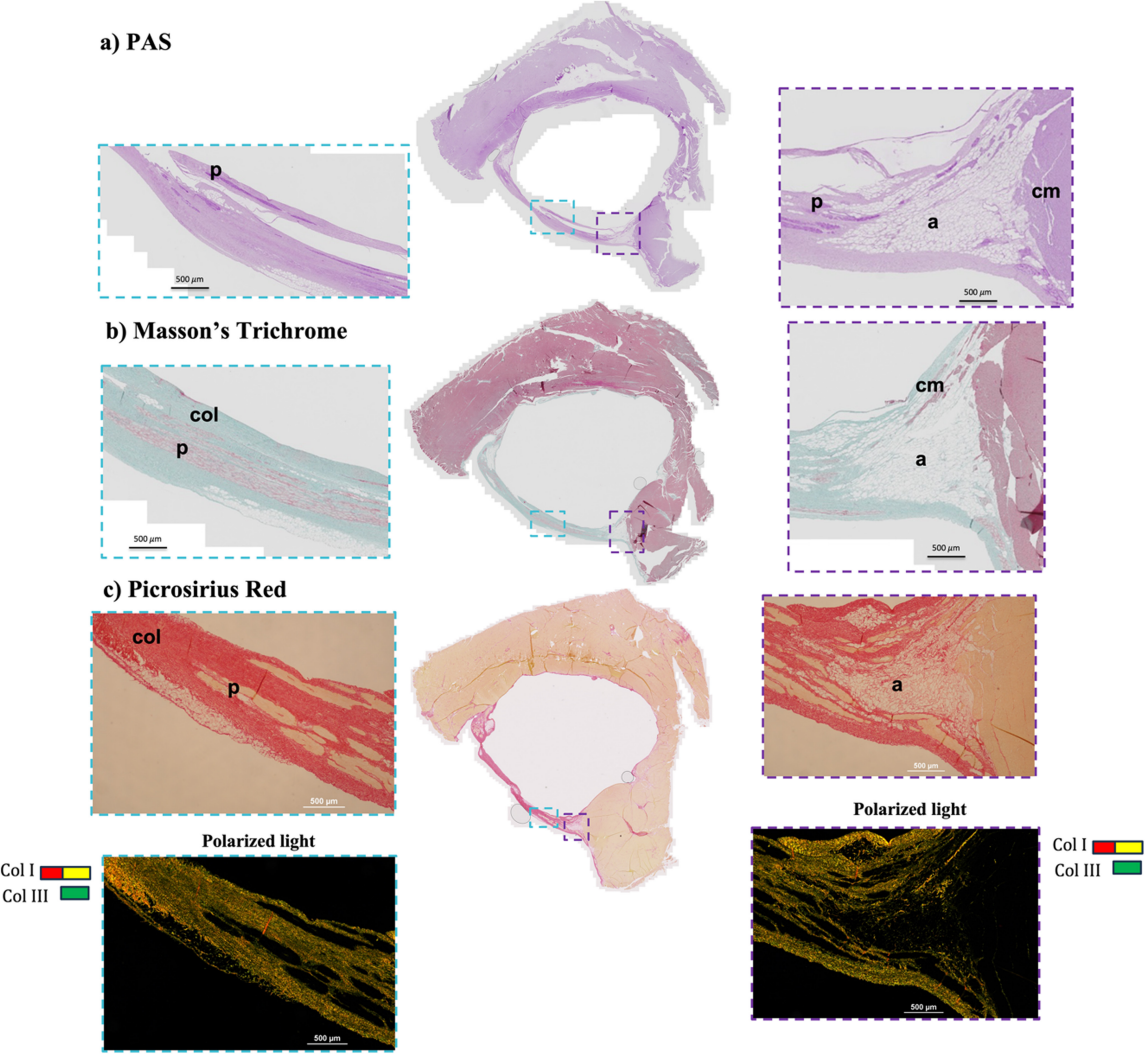
Representative histological sections, with zoomed views corresponding to the Purkinje–myocardial junction (PMJ; purple box) and free‐running (FR; light‐blue box) regions stained with (a) periodic acid–Schiff (PAS), showing darker purple signal in regions with higher glycogen content, notably in Purkinje cells; (b) Masson's trichrome, highlighting the characteristic oval morphology and larger size of Purkinje cells (as reported in sheep), with collagen stained light blue; and (c) Picrosirius red, revealing total collagen in red under normal light and distinguishing collagen types under polarized light (Type I appearing red/yellow and Type III appearing green). These staining confirm the presence of Purkinje cells (marked as “p”) within the false tendons, while also demonstrating the structural complexity of the tissue, which includes adipocytes (white aggregates marked as “a”), cardiomyocytes (marked as “cm”), and collagen (marked as “col”).

Figure 4 shows representative histological sections stained with (a) PAS, (b) Masson's trichrome, and (c) Picro Sirius Red. In Masson's trichrome–stained sections, Purkinje cells are identified by their oval morphology and larger size compared with surrounding cardiomyocytes in sheep [33, 34, 35]. PAS staining reveals increased glycogen content, which is particularly prominent in Purkinje cells. Picro Sirius Red staining highlights total collagen in red under normal illumination, while polarized light microscopy allows differentiation between collagen Type I (red, orange, and yellow) and collagen Type III (green). White aggregates observed in the images correspond to adipocytes.

**FIGURE 5:**
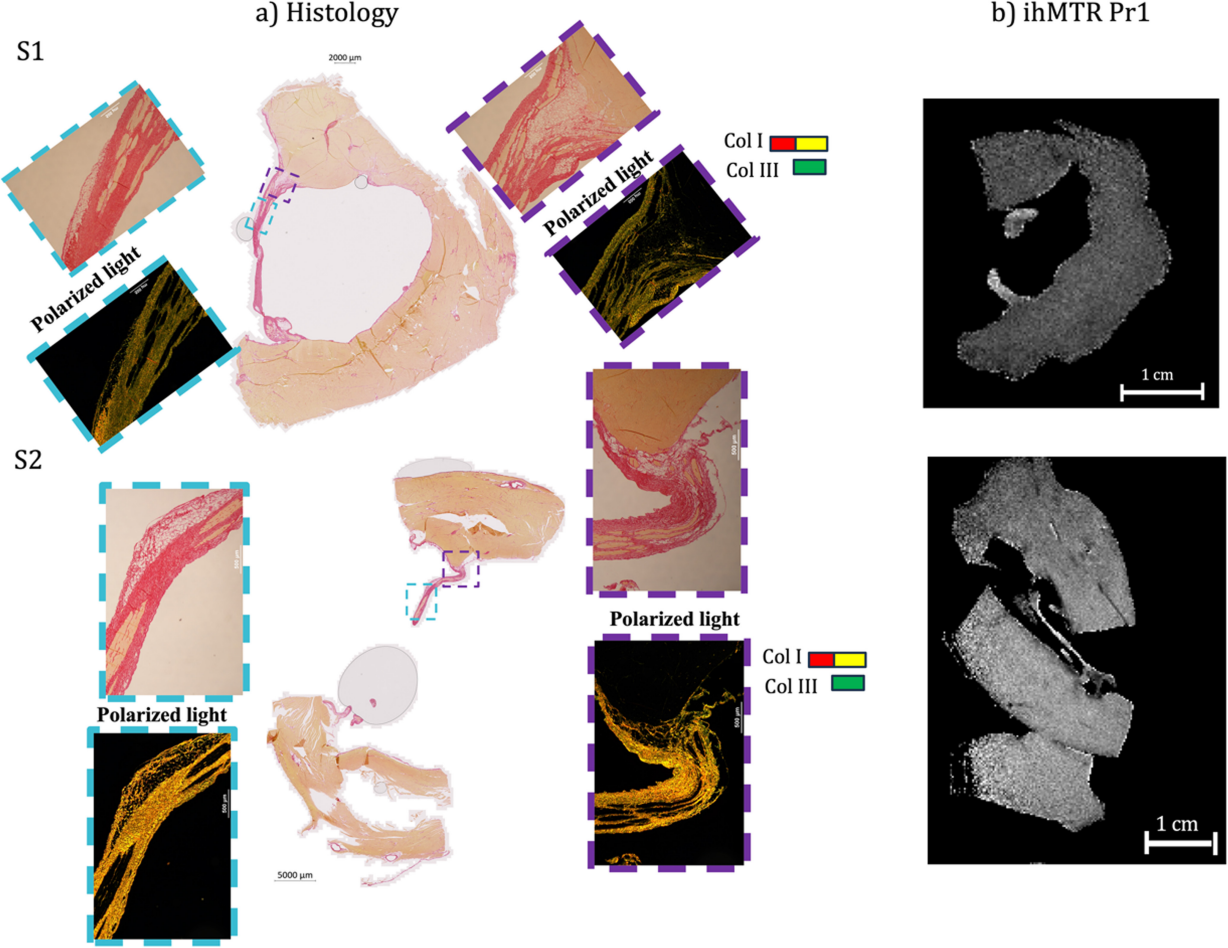
(a) Histology images with Picro Sirius Red staining best matching the ihMTR image slice of two representative samples. The polarized light (PL) filtered images depict collagen Type I in red and yellow, whereas collagen Type III appears in green. The light blue rectangle indicates the free‐running and its corresponding zoomed‐in images; the purple rectangle highlights additional zoomed‐in views of the PMJ. (b) The ihMTR map from Pr1 is also shown for comparison.

Figure 5 presents histological sections with Picro Sirius Red staining (a) best matching the corresponding acquired ihMTR image (b) for two representative samples. The histological sections reveal the detailed architecture of the fibers, including Purkinje cells, collagen, adipocytes, and cardiomyocytes.

PFs show a complex microstructure, with Purkinje cells surrounded by collagen (stained red under standard light) and adipocytes (visible as white aggregates). In each histological image, purple rectangles indicate zoomed‐in views of the PMJ, highlighting the predominance of adipocytes in these regions. In contrast, light blue rectangles mark the free‐running fiber regions, which are primarily enriched in collagen. These distinct structural patterns further support the differentiation between the PMJ and free‐running subregions of the Purkinje network.

Table 4 presents the mean ± SD % of total collagen, adipocytes, collagen Type I, and collagen Type III for each sample, averaged across multiple high‐magnification images acquired from the free‐running and PMJ regions and across multiple microscope slides. A paired *t*‐test over all samples revealed a significant difference in total collagen content between the free‐running and PMJ ROIs (72.4% ± 15.9% vs. 31.1% ± 13.1%, respectively; *p* = 0.001). In contrast, adipocyte content is lower in the free‐running region than in the PMJ, although this difference did not reach statistical significance. PL imaging indicates an overall predominance of collagen Type I over collagen Type III within the fibers (see Table 4). Collagen Type I content is slightly higher in the free‐running region compared with the PMJ (93.2% ± 3.1% vs. 85.7% ± 5.1%), whereas collagen Type III content is modestly higher in the PMJ than in the free‐running region (8.8% ± 3.9% vs. 3.0% ± 2.8%).

**TABLE 4:** Mean ± SD in % of total collagen, total adipocytes, collagen Type I, and collagen Type III for each sample, averaged across multiple images and microscope slides. The table reports both per‐sample values and averages across all samples. A paired *t*‐test indicates a significant difference in total collagen percentage between the free‐running and PMJ ROIs (*p*‐value = 0.001).

	% Total col		% Adp		% Col Type I		% Col Type III	
Sample	Free‐running	PMJ	Free‐running	PMJ	Free‐running	PMJ	Free‐running	PMJ
1	84.3 ± 12.3	44.7 ± 19.2	5.7 ± 3.8	17 ± 7.9	93.5 ± 2.1	87.3 ± 6.3	2.2 ± 2.3	8.6 ± 6.4
2	77.7 ± 9.3	45.5 ± 3.4	1.8 ± 4.9	8.5 ± 4.9	96.4 ± 1.1	93.1 ± 1.4	0.4 ± 0.7	3 ± 1.3
3	87.7 ± 7.3	25.1 ± 14.2	0.1 ± 0.1	20 ± 8.2	95.7 ± 1.2	80 ± 18.2	0.5 ± 0.4	9 ± 5.9
4	49.4 ± 13.6	16.8 ± 5.2	6.4 ± 3.2	11.4 ± 7.3	88.7 ± 5.3	86.2 ± 4.7	7.1 ± 6	9.4 ± 5.3
5	62.9 ± 7.5	23.4 ± 7.2	5.1 ± 5.1	4.8 ± 2.6	91.7 ± 2.6	81.7 ± 7.9	4.6 ± 2.9	14.1 ± 8.2
Mean ± SD	72.4 ± 15.9	31.1 ± 13.1	3.8 ± 2.7	12.3 ± 6.1	93.2 ± 3.1	85.7 ± 5.1	3.0 ± 2.8	8.8 ± 3.9

## 4 Discussion

This study provides a proof of concept for the application of the ihMT technique to experimentally image the CCS, specifically the PFs. Imaging the His–Purkinje system presents significant challenges due to its fine anatomical structure, which requires high spatial resolution imaging and has distinct functional properties. To date, the most effective method for visualizing this network has been micro‐CT with contrast agents applied to fixed cardiac samples [8, 10]. Although micro‐CT offers high spatial resolution and excellent visualization, its main limitation lies in the extensive sample preparation required, typically involving fixation, dehydration, and staining with contrast agents, which can damage tissue and prevent subsequent histological analysis, and in addition, is not transferable to in vivo imaging. MR microscopy at 17.6 T combined with 3D volume rendering enables noninvasive tracing of electrical conduction pathways from the bundle branches to the PF network [13]. Diffusion tensor imaging (DTI) MRI, which is sensitive to water mobility, has been used to characterize the microstructural orientation of cardiomyocytes and sheetlets in rabbit hearts across different mechanical states through high‐resolution tractography [36]. In contrast, our ihMT approach is sensitive to macromolecular organization, suggesting a complementary contrast mechanism for characterizing the CCS.

Our study demonstrates that ihMT offers a unique contrast mechanism for imaging PFs, revealing their microstructural composition. By testing various ihMT saturation configurations on one sample, we identified three protocols that yielded the highest signal in fibers as well as high contrast between different regions of fiber and myocardium. In general, short pulse repetition times (∆*t*), high *B*_1_ saturation power, and frequency offset in the order of 18 and 20 kHz are more suitable for imaging cardiac tissues. Conversely, long ∆*t* values (3 ms) produced low ihMTR signals. This is expected from the theory of ihMT and is attributed to the filtering of short *T*_1D_ components, as well as the amplitude loss of signal coming from longer *T*_1D_s [37]. Increasing *B*_1_ should in principle increase ihMTR values. However, even though specific absorption rate (SAR) regulations are less of a constraint in preclinical imaging as compared to human MRI, the hardware itself (power amplifier, RF coils, etc.) may become a limiting factor when the RF power approaches their tolerance limits, resulting in constraints in the maximal applied *B*_1_ RF excitation field strength that can be safely applied. In our study, we limited the *B*_1RMS_ to approximately 9.0 μT (with a maximum *B*_1peak_ at 46.4 μT), balancing safety requirements with the need for adequate signal excitation.

Notably, using the three optimized protocols, we identified that the ihMTR signal varied along the length of the PFs depending on anatomical location, whether in free‐running segments or at PMJ. These findings are consistent with previously reported structural distinctions between the two regions [7, 38]. The free‐running fibers, often referred to as false tendons, are composed primarily of conductive Purkinje cells surrounded by collagen and are specialized for rapid electrical conduction. In contrast, the PMJ are the terminal sites where impulses are transmitted from the PFs to the slower conducting working myocardium [7]. These regions may also include an intermediate layer of transitional cells that facilitate signal propagation between the two tissue types [38]. Notably, ihMT allows us to indirectly image PFs by targeting the collagen sheath surrounding the fibers.

### 4.1 Effect of Fiber Composition

This heterogeneity was further confirmed by histological analysis, which revealed distinct variations in tissue composition along the fibers. Specifically, the PMJ exhibited a higher proportion of adipocytes, whereas the free‐running segments contained greater amounts of collagen. The elevated ihMTR signal in the free‐running fibers may thus be attributed to the presence of highly ordered collagen fibers, whose organized triple‐helix structure is thought to enhance MT effects. In contrast, the lower ihMTR observed at the PMJ is likely associated with the lower total collagen content in this region. Additionally, the predominance of adipocytes at the PMJ likely contributes to the reduced ihMTR signal, as adipose tissue produces negligible MT effects [39]. Moreover, slightly higher levels of collagen Type III were observed in the PMJ region, which may contribute to the observed contrast. However, it is important to note that the ihMTR measured within each ROI, particularly at the PMJ, reflects a combination of these different tissue structures and is strongly influenced by partial volume effects.

It is also important to note that the molecular composition of PFs varies significantly across species, including sheep, pigs, rabbits, and humans [40, 41]. For instance, the study by Magat et al. [14] on porcine samples reported a markedly lower presence of adipocytes and a higher proportion of collagen Type III in the PMJ of PFs compared with the sheep samples examined in the present study. Additionally, PFs can differ across species in terms of cell size and shape, as well as the amount and organization of surrounding connective tissue [41]. These species‐specific differences in fiber composition should be carefully considered when extrapolating findings to human physiology and clinical applications.

### 4.2 Effect of Fixation and Storage

Interestingly, the average ihMT ratio in the myocardium showed considerable variability among the samples analyzed, in contrast to the fiber region, which exhibited less variability and better reproducibility. This disparity could arise from multiple factors. For instance, tissue fixation, which is known to alter water diffusion properties of biological tissues [42], may have contributed to these differences. Although the fixation protocol was standardized for all samples, variations in the time elapsed between euthanasia and fixation, as well as the duration of the fixation process, could have impacted tissue properties. Finally, intersample variability could reflect intrinsic biological differences among the sheep. It may also result from samples with free‐running fibers being taken from slightly different regions of the left ventricle.

### 4.3 Effect of Temperature and Orientation

Temperature variations have a significant effect on ihMTR values within the PF, with an estimated increase of approximately 0.29% in absolute ihMTR scale per 1°C rise in temperature (Figure S1). In contrast, the myocardium exhibited no significant changes in ihMTR with temperature variation, suggesting a tissue‐specific sensitivity to thermal conditions. In the experiments in the protocol application phase, the temperature was maintained ±1°C around 36°C, hence limiting ihMTR variations to ±0.3% (absolute value), thereby ruling out the observed ihMTR differences being due to a temperature effect.

There was a temperature difference between the protocol selection phase and the protocol application phase due to inefficiencies in the water bath system. However, as demonstrated by the temperature effect experiment, the absolute ihMTR variation is expected to be less than 1% for a 3°C change under free‐running conditions. Therefore, this temperature difference did not warrant repeating the entire protocol optimization process. Moreover, the reliability of the temperature sensor was validated (Figure S2).

Additionally, the orientation of the fibers relative to the main magnetic field (*B*_0_) did not significantly influence ihMTR values in either the PF or the myocardium (Table S2). This indicates that, under our experimental conditions, fiber orientation is not a major confounding factor in the quantification of ihMTR.

## 5 Conclusion

Despite the limited sample size and the use of 2D single‐slice imaging, this ex vivo high‐resolution study demonstrates the potential of ihMT as a novel contrast mechanism to investigate the microstructural composition of PFs. The findings suggest that ihMT may reflect the subtle differences in tissue structure, including the distribution of adipocytes, collagen, Purkinje cells, and cardiomyocytes along the fibers. Moreover, this study lays the groundwork for developing biophysical models to interpret ihMT signals in terms of tissue composition and microstructure, providing a foundation for future studies aimed at quantifying tissue properties.

Future studies should aim to validate these findings on a larger sample size, explore 3D imaging techniques, and investigate the feasibility for in vivo applications of ihMT in cardiac imaging.

## Author Contributions

**Arash Forodighasemabadi:** conceptualization, methodology, software, validation, formal analysis, data curation, writing – original draft, writing – review and editing, visualization. **Evgenios N. Kornaropoulos:** conceptualization, writing – review and editing. **Marion Constantin:** methodology, formal analysis, data curation, writing – review and editing. **Lucas Soustelle:** methodology, software, writing – review and editing. **Fanny Vaillant:** methodology, formal analysis, writing – review and editing. **Jude Leury:** methodology, software, writing – review and editing. **Richard Walton:** formal analysis, writing – review and editing. **Olivier Bernus:** formal analysis, writing – review and editing. **Bruno Quesson:** formal analysis, writing – review and editing, supervision. **Olivier M. Girard:** conceptualization, methodology, formal analysis, writing – review and editing, supervision. **Guillaume Duhamel:** conceptualization, methodology, formal analysis, writing – review and editing, supervision. **Julie Magat:** conceptualization, methodology, formal analysis, writing – review and editing, supervision, project administration, funding acquisition.

## Funding

This study received financial support from the French Government by the National Research Agency (ANR; SYNATRA ANR‐21‐CE19‐0014‐01) and Région Nouvelle Aquitaine (convention N°AAPR2022‐2021‐16609210).

## Conflicts of Interest

The authors declare no conflicts of interest.

## Supporting information


**Table S1:** ihMTR values (mean ± intra‐ROI SD) in % for each ROI, protocol, and sample.
**Table S2:** ihMTR (mean ± SD) in % in PF and myocardium for each protocol for Sample 1 oriented parallel and perpendicular to B0. The Wilcoxon signed‐rank test shows no significant differences between the ihMTR from the two different orientations.
**Figure S1:** Plot of mean ± SD of ihMTR in % as a function of temperature in free‐running (red) and myocardium (green).
**Figure S2:** Scatter plots showing the temperature in free‐running and myocardium evaluated from thermometry, as well as the temperature measured by the sensor, before and after the start of the ihMT sequence.

## Data Availability

The data that support the findings of this study are available from the corresponding author upon formal request.

## References

[1] M. Haissaguerre , E. Vigmond , B. Stuyvers , M. Hocini , and O. Bernus , “Ventricular Arrhythmias and the His–Purkinje System,” Nature Reviews. Cardiology 13, no. 3 (2016): 155–166, 10.1038/nrcardio.2015.193. 26727298

[2] A. Atkinson , S. Inada , J. Li , et al., “Anatomical and Molecular Mapping of the Left and Right Ventricular His‐Purkinje Conduction Networks,” Journal of Molecular and Cellular Cardiology 51, no. 5 (2011): 689–701, 10.1016/j.yjmcc.2011.05.020. 21741388

[3] H. E. Çetingül , G. Plank , N. A. Trayanova , and R. Vidal , “Estimation of Local Orientations in Fibrous Structures With Applications to the Purkinje System,” IEEE Transactions on Biomedical Engineering 58, no. 6 (2011): 1762–1772, 10.1109/TBME.2011.2116119. 21335301 PMC3130018

[4] F. Bogun , E. Good , S. Reich , et al., “Role of Purkinje Fibers in Post‐Infarction Ventricular Tachycardia,” Journal of the American College of Cardiology 48, no. 12 (2006): 2500–2507, 10.1016/j.jacc.2006.07.062. 17174189

[5] V. Garcia‐Bustos , R. Sebastian , M. Izquierdo , P. Molina , F. J. Chorro , and A. Ruiz‐Sauri , “A Quantitative Structural and Morphometric Analysis of the Purkinje Network and the Purkinje–Myocardial Junctions in Pig Hearts,” Journal of Anatomy 230, no. 5 (2017): 664–678, 10.1111/joa.12594. 28256093 PMC5382594

[6] E. D. Canale , G. R. Campbell , J. J. Smolich , and J. H. Campbell , Cardiac Muscle, vol. 2 (Springer Science & Business Media, 2012).

[7] D. Romero , O. Camara , F. Sachse , and R. Sebastian , “Analysis of Microstructure of the Cardiac Conduction System Based on Three‐Dimensional Confocal Microscopy,” PLoS ONE 11, no. 10 (2016): e0164093, 10.1371/journal.pone.0164093. 27716829 PMC5055359

[8] R. S. Stephenson , A. Atkinson , P. Kottas , et al., “High Resolution 3‐Dimensional Imaging of the Human Cardiac Conduction System From Microanatomy to Mathematical Modeling,” Scientific Reports 7, no. 1 (2017): 7188, 10.1038/s41598-017-07694-8. 28775383 PMC5543124

[9] A. J. Aminu , W. Chen , Z. Yin , et al., “Novel Micro‐Computed Tomography Contrast Agents to Visualise the Human Cardiac Conduction System and Surrounding Structures in Hearts From Normal, Aged, and Obese Individuals: Iodine and Graphene Oxide—Visualising Human Conduction System in Normal, Aged, and Obese Hearts,” Translational Research in Anatomy 27 (2022): 100175, 10.1016/j.tria.2022.100175.

[10] R. S. Stephenson , J. Rowley‐Nobel , C. B. Jones , et al., “Morphological Substrates for Atrial Arrhythmogenesis in a Heart With Atrioventricular Septal Defect,” Frontiers in Physiology 9 (2018): 9, 10.3389/fphys.2018.01071. 30190677 PMC6115687

[11] R. S. Stephenson , M. R. Boyett , G. Hart , et al., “Contrast Enhanced Micro‐Computed Tomography Resolves the 3‐Dimensional Morphology of the Cardiac Conduction System in Mammalian Hearts,” PLoS ONE 7, no. 4 (2012): e35299, 10.1371/journal.pone.0035299. 22509404 PMC3324466

[12] W. Chen , M. Kuniewicz , A. J. Aminu , et al., “High‐Resolution 3D Visualization of Human Hearts With Emphases on the Cardiac Conduction System Components—A New Platform for Medical Education, Mix/Virtual Reality, Computational Simulation,” Front Med (Lausanne) 12 (2025): 12, 10.3389/fmed.2025.1507005. PMC11878103 40041464

[13] M. Hwang , K. Odening , O. Ziv , B. R. Choi , G. Koren , and J. R. Forder , “Cardiac Purkinje Fiber Imaging: The First Instance of In Situ Visualization of the Conduction Path Using MR Microscopy. Proc Intl Soc Mag Reson Med,” Published online (2010).

[14] J. Magat , A. Fouillet , M. Constantin , et al., “3D Magnetization Transfer (MT) for the Visualization of Cardiac Free‐Running Purkinje Fibers: An Ex Vivo Proof of Concept,” Magnetic Resonance Materials in Physics, Biology and Medicine 34, no. 4 (2021): 605–618, 10.1007/s10334-020-00905-w. PMC8338918 33484367

[15] Y. Li , V. Casula , J. Karjalainen , M. J. Nissi , and T. Liimatainen , “Quantitative Magnetic Resonance Imaging of the Atrioventricular Conduction Axis Based on the Rotating Frame Relaxation Maps—Comparison With T2, T1 and Magnetization Transfer,” Heart Rhythm 22 (2025): 3189–3198, 10.1016/j.hrthm.2025.06.040. 40581237

[16] D. Laurent , J. Wasvary , J. Yin , M. Rudin , T. C. Pellas , and E. O'byrne , “Quantitative and Qualitative Assessment of Articular Cartilage in the Goat Knee With Magnetization Transfer Imaging,” Magnetic Resonance Imaging 19 (2001): 1279–1286, 10.1016/S0730-725X(01)00433-7. 11804755

[17] C. Wang , W. Witschey , A. Goldberg , M. Elliott , A. Borthakur , and R. Reddy , “Magnetization Transfer Ratio Mapping of Intervertebral Disc Degeneration,” Magnetic Resonance in Medicine 64, no. 5 (2010): 1520–1528, 10.1002/mrm.22533. 20677229 PMC5585868

[18] K. Jiang , C. M. Ferguson , B. Ebrahimi , et al., “Noninvasive Assessment of Renal Fibrosis With Magnetization Transfer MR Imaging: Validation and Evaluation in Murine Renal Artery Stenosis,” Radiology 283, no. 1 (2017): 77–86, 10.1148/radiol.2016160566. 27697008 PMC5359024

[19] B. C. Fuchs , H. Wang , Y. Yang , et al., “Molecular MRI of Collagen to Diagnose and Stage Liver Fibrosis,” Journal of Hepatology 59, no. 5 (2013): 992–998, 10.1016/j.jhep.2013.06.026. 23838178 PMC3805694

[20] K. López , R. Neji , R. K. Mukherjee , et al., “Contrast‐Free High‐Resolution 3D Magnetization Transfer Imaging for Simultaneous Myocardial Scar and Cardiac Vein Visualization,” Magnetic Resonance Materials in Physics, Biology and Medicine 33, no. 5 (2020): 627–640, 10.1007/s10334-020-00833-9. PMC7502043 32078075

[21] G. Varma , G. Duhamel , C. De Bazelaire , and D. C. Alsop , “Magnetization Transfer From Inhomogeneously Broadened Lines: A Potential Marker for Myelin,” Magnetic Resonance in Medicine 73, no. 2 (2015): 614–622, 10.1002/mrm.25174. 24604578 PMC4378005

[22] O. M. Girard , V. H. Prevost , G. Varma , P. J. Cozzone , D. C. Alsop , and G. Duhamel , “Magnetization Transfer From Inhomogeneously Broadened Lines (ihMT): Experimental Optimization of Saturation Parameters for Human Brain Imaging at 1.5 Tesla,” Magnetic Resonance in Medicine 73, no. 6 (2015): 2111–2121, 10.1002/mrm.25330. 24962257

[23] G. Varma , O. M. Girard , V. H. Prevost , A. K. Grant , G. Duhamel , and D. C. Alsop , “Interpretation of Magnetization Transfer From Inhomogeneously Broadened Lines (ihMT) in Tissues as a Dipolar Order Effect Within Motion Restricted Molecules,” Journal of Magnetic Resonance 260 (2015): 67–76, 10.1016/j.jmr.2015.08.024. 26408956

[24] D. C. Alsop , E. Ercan , O. M. Girard , et al., “Inhomogeneous Magnetization Transfer Imaging: Concepts and Directions for Further Development,” NMR in Biomedicine 36 (2022), 10.1002/nbm.4808. 35916067

[25] S. Mchinda , G. Varma , V. H. Prevost , et al., “Whole Brain Inhomogeneous Magnetization Transfer (ihMT) Imaging: Sensitivity Enhancement Within a Steady‐State Gradient Echo Sequence,” Magnetic Resonance in Medicine 79, no. 5 (2018): 2607–2619, 10.1002/mrm.26907. 28940355

[26] A. Forodighasemabadi , G. Baucher , L. Soustelle , et al., “Spinal Cord and Brain Tissue Impairments as Long‐Term Effects of Rugby Practice? An Exploratory Study Based on T1 and ihMTsat Measures,” Neuroimage Clin 35 (2022): 103124, 10.1016/j.nicl.2022.103124. 35905667 PMC9421542

[27] G. Duhamel , V. H. Prevost , M. Cayre , et al., “Validating the Sensitivity of Inhomogeneous Magnetization Transfer (ihMT) MRI to Myelin With Fluorescence Microscopy,” NeuroImage 199, no. May (2019): 289–303, 10.1016/j.neuroimage.2019.05.061. 31141736

[28] H. Rasoanandrianina , S. Demortière , A. Trabelsi , et al., “Sensitivity of the Inhomogeneous Magnetization Transfer Imaging Technique to Spinal Cord Damage in Multiple Sclerosis,” American Journal of Neuroradiology 41, no. 5 (2020): 929–937, 10.3174/ajnr.A6554. 32414903 PMC7228162

[29] P. Cabanis , J. Magat , J. Rodriguez‐Padilla , et al., “Cardiac Structure Discontinuities Revealed by Ex‐Vivo Microstructural Characterization. A Focus on the Basal Inferoseptal Left Ventricle Region,” Journal of Cardiovascular Magnetic Resonance 25, no. 1 (2023): 78, 10.1186/s12968-023-00989-y. 38093273 PMC10720182

[30] P. A. Yushkevich , J. Piven , H. C. Hazlett , et al., “User‐Guided 3D Active Contour Segmentation of Anatomical Structures: Significantly Improved Efficiency and Reliability,” NeuroImage 31, no. 3 (2006): 1116–1128, 10.1016/j.neuroimage.2006.01.015. 16545965

[31] L. C. U. Junqueira , G. Bignolas , and R. R. Brentani , “Picrosirius Staining Plus Polarization Microscopy, a Specific Method for Collagen Detection in Tissue Sections,” Histochemical Journal 11 (1979): 447–455. 91593 10.1007/BF01002772

[32] A. M. Jorgensen , M. Varkey , A. Gorkun , et al., “Bioprinted Skin Recapitulates Normal Collagen Remodeling in Full‐Thickness Wounds,” Tissue Engineering. Part A 26, no. 9–10 (2020): 512–526, 10.1089/ten.tea.2019.0319. 31861970 PMC7249461

[33] J. R. Sommer and E. A. Johnson , “Cardiac Muscle,” Journal of Cell Biology 36, no. 3 (1968): 497–526, 10.1083/jcb.36.3.497. 5645545 PMC2107380

[34] N. Ono , T. Yamaguchi , H. Ishikawa , et al., “Morphological Varieties of the Purkinje Fiber Network in Mammalian Hearts, as Revealed by Light and Electron Microscopy,” Archives of Histology and Cytology 72, no. 3 (2009): 139–149, 10.1679/aohc.72.139. 20513977

[35] A. Yoshimura , T. Yamaguchi , H. Kawazato , N. Takahashi , and T. Shimada , “Immuno‐Histochemistry and Three‐Dimensional Architecture of the Intermediate Filaments in Purkinje Cells in Mammalian Hearts,” Medical Molecular Morphology 47, no. 4 (2014): 233–239, 10.1007/s00795-014-0069-9. 24570344

[36] I. Teh , R. A. B. Burton , D. McClymont , et al., “Mapping Cardiac Microstructure of Rabbit Heart in Different Mechanical States by High Resolution Diffusion Tensor Imaging: A Proof‐Of‐Principle Study,” Progress in Biophysics and Molecular Biology 121, no. 2 (2016): 85–96, 10.1016/j.pbiomolbio.2016.06.001. 27320383 PMC4959513

[37] A. Hertanu , L. Soustelle , A. Le Troter , et al., “T1D‐Weighted ihMT Imaging—Part I. Isolation of Long‐ and Short‐T1D Components by T1D‐Filtering,” Magnetic Resonance in Medicine 87, no. 5 (2022): 2313–2328, 10.1002/mrm.29139. 35037302

[38] J. Tranum‐Jensen , A. A. Wilde , J. T. Vermeulen , and M. J. Janse , “Morphology of Electrophysiologically Identified Junctions Between Purkinje Fibers and Ventricular Muscle in Rabbit and Pig Hearts,” Circulation Research 69, no. 2 (1991): 429–437, 10.1161/01.RES.69.2.429. 1860183

[39] E. Ercan , G. Varma , I. E. Dimitrov , et al., “Combining Inhomogeneous Magnetization Transfer and Multipoint Dixon Acquisition: Potential Utility and Evaluation,” Magnetic Resonance in Medicine 85, no. 4 (2021): 2136–2144, 10.1002/mrm.28571. 33107146 PMC7821205

[40] V. S. Elbrønd , M. B. Thomsen , J. L. Isaksen , et al., “Intramural Purkinje Fibers Facilitate Rapid Ventricular Activation in the Equine Heart,” Acta Physiologica 237, no. 3 (2023): e13925, 10.1111/apha.13925. 36606541

[41] P. Parto , M. Tadjalli , S. R. Ghazi , and M. A. Salamat , “Distribution and Structure of Purkinje Fibers in the Heart of Ostrich (*Struthio camelus*) With the Special References on the Ultrastructure,” International Journal of Zoology 2013 (2013): 1–6, 10.1155/2013/293643.

[42] P. E. Thelwall , T. M. Shepherd , G. J. Stanisz , and S. J. Blackband , “Effects of Temperature and Aldehyde Fixation on Tissue Water Diffusion Properties, Studied in an Erythrocyte Ghost Tissue Model,” Magnetic Resonance in Medicine 56, no. 2 (2006): 282–289, 10.1002/mrm.20962. 16841346

